# Continuous care intervention with carbohydrate restriction improves physical function of the knees among patients with type 2 diabetes: a non-randomized study

**DOI:** 10.1186/s12891-022-05258-0

**Published:** 2022-03-29

**Authors:** Kade S. Lyman, Shaminie J. Athinarayanan, Amy L. McKenzie, Camy L. Pearson, Rebecca N. Adams, Sarah J. Hallberg, James P. McCarter, Jeff S. Volek, Stephen D. Phinney, John P. Andrawis

**Affiliations:** 1 Department of Orthopedics, Harbor-UCLA, 1000 W Carson St, Torrance, CA 90502 USA; 2 Virta Health, 501 Folsom Street, San Francisco, CA 94105 USA; 3 Indiana University Health Arnett, Lafayette, IN USA; 4 School of Medicine, Indiana University, Indianapolis, IN USA; 5 Abbott Diabetes Care, Alameda, CA 94502 USA; 6 Department of Genetics, Washington University School of Medicine, St. Louis, MO USA; 7 Department of Human Sciences, The Ohio State University, Columbus, OH USA; 8 Torrance Memorial Physician Network, 23560 Crenshaw Blvd Unit 102, Torrance, CA 90505 USA

**Keywords:** Type 2 diabetes, Osteoarthritis, Knee function, KOOS

## Abstract

**Background:**

In a previous study, we assessed a novel, remotely monitored carbohydrate restricted diet regimen including nutritional ketosis in patients with type 2 diabetes and reported significant improvements in weight, glycemic control, abdominal fat and inflammation from baseline to 2 years. Knee outcome measures were collected as a secondary outcome in the trial. This study aims to assess the effect of this intervention on knee functional scores and to identify if changes in weight, central abdominal fat (CAF), glycemic status and high sensitivity C-reactive protein (hsCRP) were associated with its improvement.

**Methods:**

This prospective analysis included continuous care intervention (CCI, *n* = 173) and usual care (UC, *n* = 69) trial participants with type 2 diabetes that reported knee pain at baseline. Knee outcome measures included the Knee injury and Osteoarthritis Outcome Score (KOOS) pain, symptoms, activities of daily living (ADL), sports and recreation function, and knee-related quality of life subscales, and total KOOS score were assessed from baseline to 2 years. Missing data at each time point were replaced with multiple imputation under the assumption of missing at random. To assess if the primary analysis of the knee scores changed under plausible missing not at random assumptions, sensitivity analysis was also performed using pattern mixture models. In CCI, we also assessed factors associated with the improvement of knee scores.

**Results:**

In the primary analysis, CCI participants demonstrated a statistically significant improvement in total KOOS and all KOOS individual subscale scores at 1 year and maintained through 2 years as opposed to UC patients who showed no significant changes from baseline to 2 years. The significant improvement in total KOOS and its individual subscale scores from baseline to 2 years remained relatively stable in CCI in the sensitivity analysis under different missing not at random scenarios confirming the robustness of the findings from the primary analysis. Approximately 46% of the CCI participants met the 10 points minimal clinically important change at 2 years. A reduction in CAF was associated with improvement in total KOOS and KOOS ADL, while a decrease in hsCRP was associated with improvement in KOOS symptoms scores.

**Conclusion:**

A very low carbohydrate intervention including nutritional ketosis resulted in significant improvements in knee pain and function among patients with T2D. The improvements in knee function were likely secondary to a reduction in central adiposity and inflammation. Future research on the applicability of this intervention in radiographically confirmed OA patients is important.

**Trial registration:**

Clinical trial registration: NCT02519309 (10/08/2015).

**Supplementary Information:**

The online version contains supplementary material available at 10.1186/s12891-022-05258-0.

## Background

Osteoarthritis (OA) is a painful and debilitating disease process affecting roughly 54.4 million adults (22.7%) in the United States annually [1]. Knee OA is the most common form and is particularly morbid in its effects, leading to decreased mobility, difficulty performing self-care and performing usual activities of daily living [2]. Among adults with diabetes, the prevalence of OA increases to an estimated 48.1% [3]. Although the exact mechanistic link between diabetes and OA is poorly understood, diabetes is recognized as a substantial predisposing risk factor for OA and increased OA severity [4–6]. Type 2 diabetes is a chronic metabolic disorder mainly characterized by hyperglycemia and insulin resistance. It is suggested that the persistent high level of glucose in those with diabetes alter connective tissue extracellular matrix, induces cell injury and damages synovium leading to musculoskeletal disorder [5, 7]. Likewise, low-grade systemic inflammation is observed in both diabetes and OA, and it is possibly linked to the pathophysiology of these two conditions [5]. In addition to its predisposing effects on OA, diabetes is also an independent amplifier of knee osteoarthritic pain [6, 8], and its effect on pain is independent of weight [9, 10]. However, the impact of glycemic control on knee pain is debatable with some reporting an association between elevated HbA1c and knee pain [11] while others reporting no evidence of association [12].

Among nonsurgical treatment options for knee OA, weight loss is routinely recommended and has been shown to significantly improve knee pain and function [13–15]. In fact, weight loss is one of the few nonpharmacologic treatments recommended by the American Academy of Orthopaedic Surgeons in their most recent guidelines for treatment of the osteoarthritic knee [13]. The Intensive Diet and Exercise for Arthritis (IDEA) trial demonstrated a 40% reduction in symptoms related to knee OA with a > 10% decrease in overall bodyweight [16]. It is likely that the effect of weight loss seen in these studies is due to a decrease in the knee joint loading. Specifically, thigh and abdominal fat content, in addition to overall body mass have been linked to significantly increased knee loading force [17]. Additional studies utilizing magnetic resonance imaging have also demonstrated that weight loss reduces actual overall chondral degeneration over time in patients with knee OA [18]. Likewise, the pivotal Look AHEAD trial found that weight loss leads to decreased knee pain and improved patient reported knee function scores even in overweight patients without a confirmed diagnosis of OA [19].

While the relationship between bodyweight and knee osteoarthritis has been well established, more recent studies have explored diet as an independent modulator of knee OA. Preliminary studies suggest that diet may be an independent variable influencing knee pain in osteoarthritic mice [20]. Furthermore, ketogenic diet modification has been proposed as a means of lowering overall body inflammation [21], which has been shown to be beneficial in reducing many types of inflammatory pain [22]. Among proposed mechanisms for the anti-inflammatory effects of ketosis is the inhibitory effect beta-hydroxybutyrate plays in the NLRP3 inflammasome-mediated inflammatory response [23]. Carbohydrate restriction and ketogenic diets are also widely used in the clinical management of obesity and diabetes [24–26]. Carbohydrate restriction to less than 30 to 50 g per day is often necessary to induce nutritional ketosis, defined as blood beta-hydroxybutyrate in the range of 0.5 to 3.0 mM [27–30]. Physiologically during nutritional ketosis, insulin secretion is reduced to a level that promotes fat oxidation and utilization of ketones as the main energy supply [30].

While ketogenic diets have been shown to be successful in treating diabetes and improving some forms of pain [22, 24–26], and diabetes may be an independent amplifier of knee pain [6, 8], there are currently no studies evaluating the potential independent effects of a carbohydrate restricted diet on knee pain and function in diabetes. Our recent study in patients with type 2 diabetes following a remotely monitored carbohydrate restricted diet regimen inducing nutritional ketosis reported significant improvements in weight and glycemic control from baseline to 2 years while deprescribing antihyperglycemic medication [27–29]. We also reported a significant reduction in inflammation at 2 years [27, 29]. Subjective data on patient reported knee pain and function scores were collected from these patients as a secondary outcome at each assessment time point. The knee assessment was performed with the Knee Injury and Osteoarthritis Outcome Score (KOOS) questionnaire. The KOOS is a validated patient-reported metric designed to assess the short-term and long-term symptoms and function of patients with knee injury and/or osteoarthritis [31–33]. It is a multidimensional questionnaire measuring five knee-related domains including pain, symptoms, activities of daily living, sports and recreation function and knee-related quality of life.

We postulate that a carbohydrate restriction intervention would improve knee pain and function scores and it is possibly driven by the improvement in weight, glycemic control, and inflammation. Therefore, in this study, we aim to evaluate the effect of this carbohydrate restriction intervention on subjective knee pain and function scores that were secondarily collected as part of the trial. In addition, we also assessed and identified factors that were associated with the knee improvements in the intervention. For the association analysis, we included outcome measures that were previously reported and commonly associated with knee pain and OA [16–19].

## Methods

### Study design

The full detail of the study design, inclusion and exclusion criteria and the results from primary outcomes have been previously published [32, 33]. This study was approved by the Franciscan Health Lafayette Institutional Review Board and registered under Clinical trials.gov identifier NCT02519309. Eligible participants were aged between 21 and 65 years with a diagnosis of T2D and a BMI > 25 kg/m^2^ and all participants provided written informed consent. The trial was an open-label, non-randomized, controlled, longitudinal study with patients divided into two groups: remotely monitored continuous care intervention (CCI) and usual care (UC). In the CCI regimen, participants self-selected to receive educational content either through on-site group meetings (CCI-onsite) or virtual delivery (CCI-virtual). The medical treatment and educational content were the same between the CCI-onsite and CCI-virtual, except the CCI-onsite group had additional clinic group meetings that ranged from weekly for 12 weeks to quarterly in the second year (26–28). All participants in the CCI arm were given access to a mobile health application (app) that allowed them to communicate with a team of healthcare professionals consisting of a personal health coach and health care provider (physician or nurse practitioner). These patients were educated and guided remotely to achieve nutritional ketosis, which typically included a daily dietary carbohydrate restriction to less than 30 g. Patients were also encouraged to measure body weight daily and input fingerstick blood glucose and beta hydroxybutyrate concentrations in the app allowing for continuous monitoring of patients’ diet adherence, progress and to guide their healthcare team to make appropriate adjustments. Since there were no significant differences in the knee outcomes including total KOOS and its individual subscale scores between the two education groups, the CCI-onsite and CCI-virtual groups were combined for the analyses in this study.

The usual care group was recruited separately. This group consisted of T2D patients who began participating in a local diabetes education program with treatment by their primary care physician or endocrinologist and diet counseling by registered dietitians. No modification was made to their care for the study. This group served as a reference of diabetes disease progression under routine care within the same geographical area and health system.

### Study participants in knee assessment

The recruitment of patients into the study were mainly based on the inclusion and exclusion criteria listed for the primary outcomes. There were 262 CCI and 87 UC participants with T2D enrolled at baseline for the main study. The study was not designed for assessing knee outcome; the knee subjective data were collected as a secondary outcome in the trial along with other questionnaires assessing sleep and depression symptoms. The KOOS questionnaires were administered to participants who completed their laboratory assessments by the study coordinator during their baseline, 1- and 2-years follow-up visit in the clinic. The patients completed paper-based questionnaires after their clinic visit. At baseline, there were some patients who failed to complete the questionnaire or sections of the questionnaire. At 1 and 2 years, in addition to patients not completing the questionnaire, missingness in the knee questionnaire data was also related to patients withdrawing from the study, and patients failed to attend their clinic follow-up visit after completion of their laboratory assessment.

For the knee assessment, participants who reported no knee pain at baseline (based on their response to question P1 “How often do you experience knee pain” of the KOOS questionnaire) were excluded from analysis and those who reported knee pain at baseline or missing any baseline data were retained for further analysis (Supplementary Fig. 1, 173 CCI; 141 with data and 32 missing, 69 UC; 52 with data and 17 missing). Missing data at each assessment time point were multiple imputed for the final analysis.

### Outcome measures

#### Demographic and clinical variables

Both CCI and UC participants underwent comprehensive laboratory analysis and clinical anthropometric assessment at baseline, 1 year, and 2 years. Details of the methods were previously published [27, 29]. Blood analytes were processed at a laboratory certified by the Clinical Laboratory Improvement Amendments (CLIA). CCI participants were further assessed for body composition changes at baseline, 1 and 2 years using dual X-ray absorptiometry (DXA) (Lunar GE Prodigy, Madison, WI) analyzed using GE Encore software (v11.10, Madison, WI). The data assessing safety, effectiveness, and sustainability of the two intervention arms (CCI and UC) on glycemic status, diabetes medication use, weight and other metabolic parameters were previously published. Some of these clinical variables that significantly improved in the CCI arm from baseline to 2 years [27, 29] including body weight, body composition (central abdominal fat), glycemic status (HbA1c and fasting glucose) and inflammation (high sensitivity C-reactive protein and white blood cells) were included in this study for a post-hoc association analysis in the CCI group.

### Knee injury and Osteoarthritis Outcome Score (KOOS)

Clinical assessment of knee symptoms was performed with the Knee injury and Osteoarthritis Outcome Score (KOOS) survey. The KOOS is a multi-dimensional questionnaire measuring five knee-related domains including pain, symptoms, activities of daily living, sports and recreation function, and knee-related quality of life. The KOOS and its individual sub-scores were used as a subjective measure to gauge patient knee pain and function with or without a diagnosis of knee osteoarthritis. Each section in the KOOS survey is scored on a normalized 0–100 scale (with 100 categorized as the best with no knee problems and 0 as the worse knee outcome). The total KOOS score was calculated as the averaged individual KOOS subscale scores (0–100). The Cronbach’s α coefficients between the KOOS individual sub-scores and total KOOS scores were excellent in all three time points (> 0.95).

### Statistical analyses

All data analyses were conducted using IBM SPSS Statistics for Windows, version 26.0 (IBM Corp, Armonk, N.Y., USA). We assessed the normality and linearity of the knee KOOS data including KOOS Symptoms, KOOS Pain, KOOS Activities of Daily Living (KOOS ADL), KOOS Sports and Recreation (KOOS Sport), KOOS Quality of Life (KOOS QoL) subscores and total KOOS score from all three time points using skewness and kurtosis [34].

Descriptive statistics of the baseline characteristics were performed, and independent sample student t-tests were used to analyze differences in baseline characteristics between CCI and UC, and completers and non-completers (including dropouts). The difference in the proportion of missing data at each time point between CCI versus UC was assessed using Chi-Square test.

Due to high degree of missingness in the knee outcome data. The primary assessment of KOOS total and subscores was performed under missing at random (MAR) assumption using multiply imputed dataset. A standard multiple imputation (MI) was performed to generate 50 imputed datasets. For within-group longitudinal changes from baseline to 2 years and between-group differences (CCI vs UC) at 1 and 2 years in the KOOS total and sub-scores, we conducted linear mixed-effects model (LMMs) with time as a repeated measure. The models included time, group effect (CCI vs UC) and time by group interaction as fixed effects and several baseline and demographic covariates (as listed in the Table 1 footnote). A maximum likelihood-approach was used for parameter estimation and the correlations between repeated measures were accounted for using an unstructured covariance. The LMMs were carried out for each multiple imputed datasets as an intent-to-treat analysis and the results were combined using Rubin’s rules. A second completers only analysis was also performed on participants with available data at baseline and 2 years.

**Table 1:** Adjusted mean changes over time in participants with knee pain at baseline [CCI T2D versus UC T2D]

	Baseline Mean (95% CI)	*n*	1 year Mean (95% CI),	*n*	Change from baseline (95% CI)	2 years Mean (95% CI)	*n*	Change from baseline (95% CI)
**Total KOOS**								
CCI	69.4 (66.6, 72.2)	141	79.3 (76.7,81.9)	111	9.9 (6.9, 12.5) ***	79.4 (76.7, 82.0)	103	10.0 (7.4,13.5) ***
Usual Care	64.7 (60.2, 69.2)	50	65.2 (60.9, 69.4)	57	0.5 (−4.1, 5.0)	61.0 (56.7, 65.2)	37	−3.7 (−8.7, 0.9)
CCI vs. UC	4.6 (− 0.7, 10.0)		14.1 (9.1, 19.1) ***			18.4 (13.4, 23.5) ***		
**KOOS Symptoms**								
CCI	72.8 (69.9, 75.6)	152	79.8 (77.1, 82.6)	115	7.0 (4.1, 9.8) ***	80.3 (77.2, 83.5)	107	7.5 (4.4, 10.7) ***
Usual Care	71.2 (66.2, 76.3)	51	70.2 (65.0, 75.4)	57	−1.0 (−5.7, 3.5)	65.8 (59.0, 72.7)	38	−5.4 (−0.0, − 10.9)
CCI vs. UC	1.6 (−3.5, 7.0)		9.6 (3.3, 13.1) ***			14.5 (10.0, 21.6) ***		
**KOOS Pain**								
CCI	74.7 (71.9, 77.5)	149	83.5 (80.6, 86.3)	112	8.7 (5.7, 11.8) ***	84.0 (80.7, 87.2)	107	9.3 (6.0, 12.6) ***
Usual Care	68.6 (63.9, 73.4)	52	67.4 (63.0, 71.7)	57	−1.2 (−6.3, 3.6)	67.5 (60.9, 74.0)	39	−1.1 (− 6.7, 3.3)
CCI vs. UC	6.1 (0.6, 11.3)		16.1 (11.2, 21.4) ***			16.5 (13.1, 23.5) ***		
**KOOS Activities of Daily Living**								
CCI	78.9 (76.1, 81.7)	151	87.1 (84.6, 89.5)	115	8.2 (5.4, 11.2) ***	87.4 (84.5, 90.2)	107	8.5 (5.2, 11.8) ***
Usual Care	71.9 (67.2, 76.5)	52	70.9 (67.1, 74.7)	57	−1.0 (−5.2, 3.5)	64.5 (59.8, 69.3)	39	−7.4 (−12.9, − 1.9) **
CCI vs. UC	7.0 (1.8, 12.3) **		16.2 (12.0, 21.0) ***			22.9 (17.9, 28.7) ***		
**KOOS Sport and Recs**								
CCI	58.9 (54.2, 63.7)	143	73.1 (68.7, 77.5)	114	14.2 (9.2, 19.4). ***	71.7 (66.4, 77.1)	103	12.6 (6.7, 19.1) ***
Usual Care	60.8 (53.2, 68.4)	51	61.4 (54.6, 68.1)	57	0.6 (−7.0, 8.6)	54.9 (46.3, 63.5)	38	−5.9 (− 14.3, 3.1)
CCI vs. UC	−1.9 (− 10.3, 6.6)		11.7 (3.2, 19.6) **			16.8 (7.7, 25.9) ***		
**KOOS Quality of Life**								
CCI	61.7 (57.9, 65.5)	150	73.0 (69.1, 76.9)	115	11.3 (7.4, 15.3) ***	73.6 (69.6, 77.6)	106	11.9 (8.3, 15.9) ***
Usual Care	50.9 (44.3, 57.4)	52	55.9 (49.9, 61.8)	57	5.0 (−1.4, 10.1)	52.1 (44.7, 59.5)	39	1.2 (−5.2, 7.8)
CCI vs. UC	10.8 (4.1, 18.6) **		17.1 (10.6, 24.7) ***			21.5 (14.9, 27.9) ***		

*Note*. Ns for the final analysis in continuous care intervention =173 and for usual care = 69. In the table “n” represents those with available data at each time points; any missing data at each assessment timepoints were multiple imputed 50 times. Unless otherwise noted, estimates reported were obtained from linear mixed-effects models which provide adjusted means and mean changes, controlling for baseline age, sex, race, body mass index, and insulin use for each imputed dataset. The final estimates were pooled using Rubin’s rules resulting in an intent-to-treat analysis. A significance level of *P* < 0.008 ensures overall simultaneous significance of *P* < 0.05 over the 6 variables using Bonferroni correction

*Abbreviations*: *T2D* type 2 diabetes, *CI* confidence interval, *CCI* continuous care intervention, *UC* usual care, *KOOS* knee osteoarthritis outcome scores

^***^p < 0.001

^**^p < 0.01

To further investigate the possibility of the data missing not at random (MNAR), several sensitivity analyses were conducted using pattern-mixture models (PMMs) applying different scenarios of plausible departures from missing at random (MAR) assumption. The imputed values in the 50 MI datasets were multiplied by a sensitivity parameter delta (δ) representing the average difference between the observed values collected from the completers and those missing any values. For the total KOOS and subscores data, we reported a significant increase of at least 10% from baseline in CCI in the primary analysis using MI data with MAR assumption. Based on the primary analysis, we estimated a difference of 5 to 10% is considered as a reliable δ to assess the different MNAR scenarios. A comprehensive step-by-step detailed description of the sensitivity analysis using PMMs is included in the Supplementary Method Section.

As an additional exploratory analysis only in the CCI group, we then assessed predictors associated with KOOS improvement. This analysis was performed among those who completed the study with available knee data at 2 years. We first performed unadjusted Pearsons’ correlations between changes in total KOOS and its subscale scores with changes in glycemic, weight, abdominal fat and inflammation at 1 and 2 years. Variables with significant correlations were then included in multivariate linear mixed-effects regression models with random intercepts and random time slopes. Six different models were generated to test the association between the total KOOS score and its individual subscale scores with these potential predictors. In these models, the time trend was controlled, and the predictors were modeled as a time-varying covariate. The models also included predictors by time interaction term to assess concurrent association over time between the predictor variables and KOOS. The models also included several baseline and demographic covariates (as listed in the Table 2 footnote).

**Table 2:** Regression coefficients (β), 95% confidence intervals and *p*-values for each predictor from linear mixed effects regression models associated with total and individual KOOS subscale scores

Variables	Total KOOS		KOOS Symptoms		KOOS Pain		KOOS ADL		KOOS Sport		KOOS QoL	
	β (95% CI)	***P***	β (95% CI)	***P***	β (95% CI)	***P***	β (95% CI)	***P***	β (95% CI)	***P***	β (95% CI)	***P***
**CAF**	**−5.0 (−9.8, −0.2)**	**0.04**	−4.1 (−8.7,5.0)	0.08	−3.4 (−7.9,1.2)	0.15	**−4.8 (−9.3, −0.3)**	**0.04**	−4.8 (−11.8, 2.2)	0.18	−4.6 (− 10.3, 1.2)	0.12
**Weight**	0.0 (−0.2,0.0)	0.81	−0.0 (− 0.2,0.2)	0.89	− 0.0 (− 0.2,0.2)	0.98	0.0 (− 0.1,0.2)	0.83	0.0 (− 0.1,0.3)	0.86	−0.0 (− 0.2, 0.2)	0.91
**hsCRP**	−0.8 (− 1.6,0.1)	0.08	**− 0.9 (− 1.8, − 0.1)**	**0.03**	−0.8 (− 1.6,0.1)	0.08	−0.7 (− 1.6,0.1)	0.08	−0.7 (−2.0,0.5)	0.26	− 0.8 (− 1.8, 0.2)	0.1

*Note*. Ns for continuous care intervention = 103, completers with available knee score data at 2 years. The models included all three predictors and their interaction terms. The time was treated as a random slope. Baseline age, sex, race, body mass index, and insulin use were used as covariates in the models. This maximum likelihood-based approach uses all available data

Additionally, to further explore clinical improvement, we assessed the efficacy of the interventions using minimal clinically important change (MIC) at 2 years among those who completed the study with available knee data at baseline and 2 years. We utilized a cut-off of 10 (31,33) for MIC; those achieving a > + 10 increase in total KOOS score from baseline to 2 years were classified as “improvement” and those having a lower score or < 10 increase in score were classified as “no improvement”. Proportions of participants with improvement and no improvement were calculated in both CCI and UC and between-group differences were analyzed using logistic regression adjusted for age, gender, race, and diabetes duration. Factors associated with likelihood of achieving “improvement” versus “no improvement” in CCI were assessed with logistic regression.

For the primary aim of the paper assessing longitudinal changes in the total and individual subscale scores, a Bonferroni adjusted *p*-value =0.05/6 variables = 0.008 was used to determine statistical significance. For all other exploratory analyses, a p-value = 0.05 was used to determine statistical significance.

## Results

### Baseline participants characteristics

Patient baseline characteristics for knee scores and other demographic, metabolic and inflammatory variables among CCI and UC participants reporting knee pain at baseline are provided in supplementary Table 1. There was a total of 173 CCI and 69 UC participants reporting knee pain at baseline. Participant groups were consistent with each other in their baseline demographic, biochemical variables, and total KOOS and subscale scores, except for body weight and white blood cell count (Supplementary Table 1). However, using standardized differences there were 8 variables showing imbalance between CCI and UC participants including age, gender, diabetes duration, weight, white blood cell count, KOOS pain, KOOS activities of daily living and KOOS quality of life. There were no significant differences between completers and non-completers in the baseline total KOOS and subscale scores in CCI and UC, except that KOOS QoL was significantly greater in non-completers versus completers in UC.

### Missing data

Missing data were encountered in all assessment time points even at baseline in both the intervention arms (Supplementary Fig. 1). The greatest level of missingness was observed at 2 years where only 59.5% of CCI and 53.6% of UC participants had complete KOOS data. Missing data at 2 years were mainly due to patients who had withdrawn from the study (28.9% in CCI and 23.2% in UC) and patients missed the follow-up clinic appointment or patients failed to complete the questionnaire (12.1% in CCI and 23.2% in UC). The distribution of missing data was consistent between CCI and UC at baseline (18.5% in CCI vs 27.5% in UC) and 2 years (40.5% in CCI and 46.4% in UC) but at 1-year, missing data were more common in CCI than UC (35.8% in CCI vs 17.4% in UC, *p* = 0.003).

### Primary post-intervention analysis in total KOOS and subscale scores

**Fig. 1:**
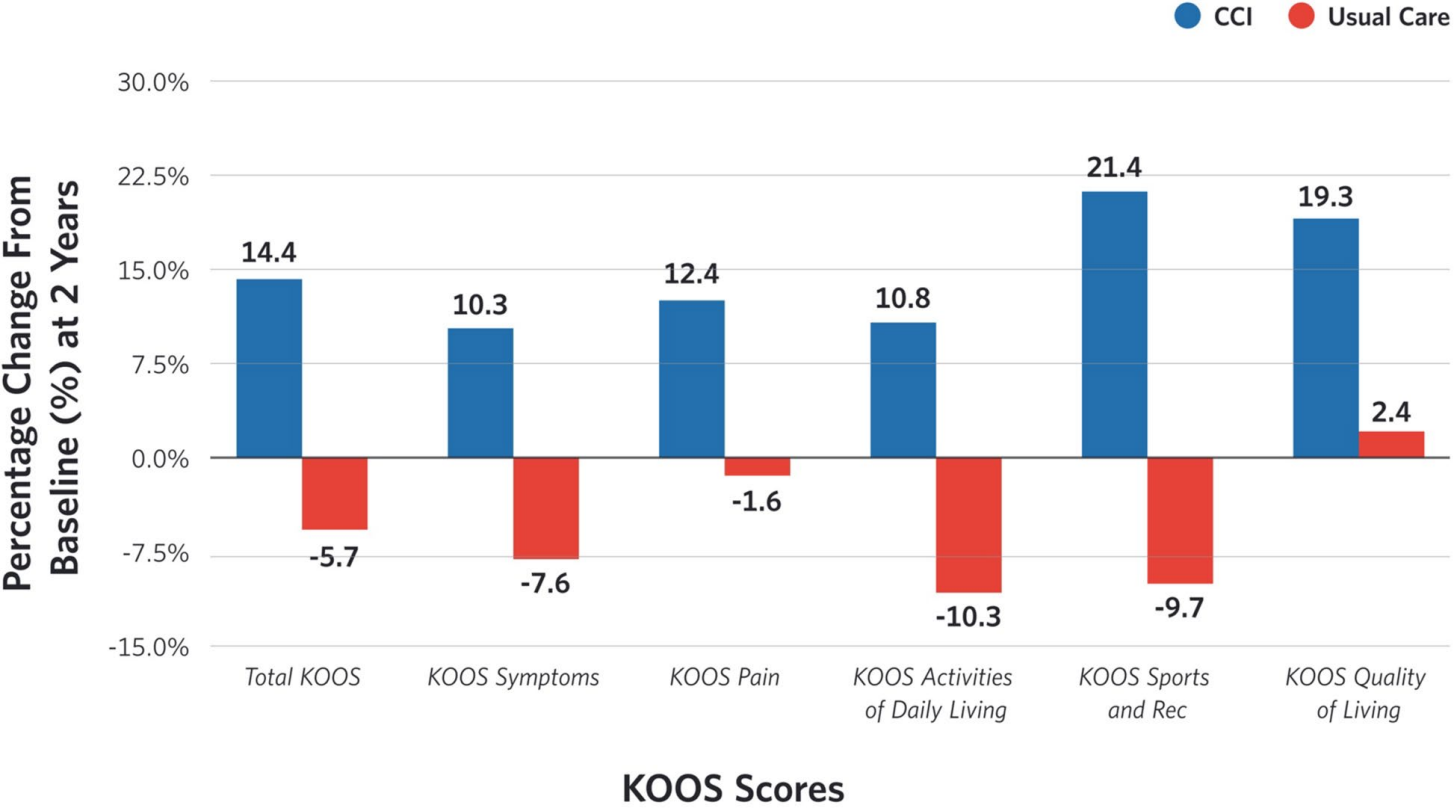
Percentage changes in the total and its individual subscale Knee Injury and Osteoarthritis Scores (KOOS) from baseline to 2 years in Continuous Care Intervention and Usual Care

Among participants with knee pain at baseline (CCI, *n* = 173; UC, *n* = 69), CCI demonstrated a statistically significant improvement in total KOOS and all KOOS individual subscale scores at 1 year compared to UC patients [Table 1; total KOOS increased at 1 year = 9.9 (95% CI: 6.9, 12.5) in CCI, decreased in UC = − 0.5 (95% CI: − 4.1, 5.0)]. These improvements remained significant from baseline at 2 years [Table 1; CCI = 10.0 (95% CI: 7.4, 13.5) increase, UC = − 3.7 (95% CI: − 8.7, 0.9) decrease) with no significant differences between 1 and 2 years. There were approximately 10% increase in total KOOS and its subscale scores at 1 and 2 years (Fig. 1) in CCI. Similar improvements were not observed in UC (Fig. 1). The overall mean improvement in total KOOS and its subscale scores in CCI at 1 and 2 years also reached the minimal clinically important change of 8–10 points, except for KOOS Symptoms (7.0 at 1 year and 7.5 at 2 years).

The CCI group also demonstrated significantly higher scores compared to their UC counterparts at 1 year (*p* < 0.01) and 2 years (*p* < 0.01) in total KOOS as well as in all KOOS subscales (Table 1). Results of completers only were consistent with the intent-to-treat analysis.

### MNAR sensitivity analysis

**Fig. 2:**
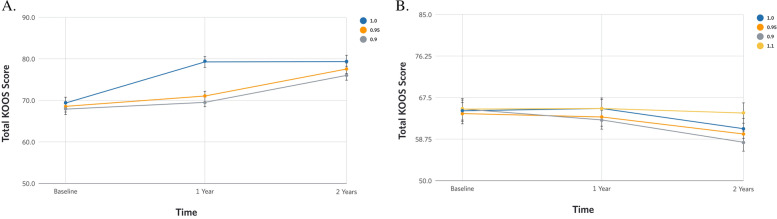
Longitudinal change in total Knee Injury and Osteoarthritis Scores (KOOS) from baseline to 2 years under different MNAR scenarios. **A**. Continuous Care Intervention. 1: Primary imputed values under missing at random assumption. 0.95: Primary imputed values estimated 5% lower. 0.90: Primary imputed values estimated 10% lower. **B**. Usual Care. 1: Primary imputed values under missing at random assumption. 0.95: Primary imputed values estimated 5% lower. 0.90: Primary imputed values estimated 10% lower. 1.10: Primary imputed values estimated 10% higher

The longitudinal changes in KOOS total and its subscores under different MNAR modified missing data assumption are listed in Supplementary Table 2. The PMM sensitivity analysis demonstrated that the longitudinal changes in KOOS total and its subscores remain relatively stable in both CCI and UC under different MNAR assumption (MAR departures). In all the MAR departure scenarios, the CCI showed significant improvements in total KOOS (Fig. 2A) and its subscores from baseline to 2 years (Supplementary Table 2) but not in UC (Fig. 2B, Supplementary Table 2) as originally demonstrated in the primary MI dataset under MAR assumption. For example, even when the missing values were estimated to be 10% lower in CCI and 10% higher in UC, we observed similar trend of overtime change in total KOOS and its subscores in both CCI and UC. This confirms that the missing pattern between the two treatment groups CCI and UC are similar where both groups equally experience MNAR bias that cancels out when we performed the sensitivity analysis. This is further corroborated by the equal distribution of missing data or attrition between CCI and UC at baseline and 2 years.

### Identifying factors associated with knee score improvement in CCI

Unadjusted Pearson correlation revealed significant inverse correlations between change in weight, central abdominal fat (CAF) and hs C-reactive protein (hsCRP) with change in total KOOS and several of the KOOS subscale scores. Changes in HbA1c, fasting glucose, and white blood cell were not correlated with changes in total KOOS and its individual subscale scores.

We then performed multivariate linear mixed-effects regression models that included all three potential predictors: weight, CAF and hsCRP with random intercepts and random time slopes. CAF was the only factor significantly and negatively associated with improvement of total KOOS (β= − 5.0, *p* = 0.04) and KOOS ADL (β= − 4.8, p = 0.04) scores, where a decrease in CAF was associated with higher total KOOS and KOOS ADL scores (Table 2). There were no significant associations between weight, CAF, or hsCRP with change in KOOS pain, sport and quality of life. However, for KOOS symptoms, hsCRP was the only factor significantly associated with its improvement with β= − 0.9 (p-0.03), where a decrease or lower hsCRP was associated with higher KOOS Symptom score (Table 2).

### KOOS Minimal Clinically Important Change (MIC)

At 2 years, 45.5% of the CCI participants and 14.8% of UC participants were classified as total KOOS score ‘improvement”. As an exploratory analysis, the odds of achieving the MIC in total KOOS was 5.4 times (95% CI: 1,7, 17.9, *p* = 0.01) greater in CCI than UC and greater reduction in both CAF and hsCRP at 2 years were associated with greater likelihood of achieving the MIC (CAF, OR = 0.11, *p* = 0.02 and hsCRP, OR = 0.79, *p* = 0.03) in CCI.

## Discussion

The purpose of this secondary analysis was to evaluate the effect of a novel, remotely monitored carbohydrate restricted dietary intervention including nutritional ketosis on subjective knee pain and function scores in patients with type 2 diabetes (T2D), and to identify factors associated with improvement. In this paper, we also conducted sensitivity analysis using pattern mixture-models (PMMs) to assess the issue of missingness in our data and the likelihood of the data missing not at random (MNAR) [35, 36]. Missingness is a common issue encountered in patient reported outcome measures especially when the data is collected as a secondary endpoint [37] and if paper-based data collection is used [38, 39].

Our primary analysis results suggest that a medically supervised carbohydrate restricted diet and continuous care intervention statistically and clinically improved total KOOS and all its individual subscores at 1 year and that these improvements are largely maintained out to 2 years among patients with knee pain. In contrast, knee function and pain in the usual care arm did not change significantly from baseline showing a gradually worsening trend commonly observed in patients with T2D [4, 5, 40]. In accordance with the primary analysis, our MNAR sensitivity analysis using PMMs also confirmed that the continuous care intervention showed a statistically significant longitudinal improvement in total KOOS and all its individual subscores from baseline to 2 years even when we assumed a differential attrition pattern between CCI (those missing data had worse outcomes) and UC (those missing data had better outcomes) [36, 41]. The sensitivity analysis confirms that the primary post-intervention analysis for total KOOS and its subscores can be interpreted as robust to MNAR assumption and the overall interpretation of the primary analysis did not change [36, 41]. Our findings were consistent with a recent study that reported a significant decrease in pain scores for patients with knee OA via a low carbohydrate diet compared to a low fat and a routine diet [42].

Compared to prior publications, while our analysis demonstrates that weight reduction correlated with improvement in overall KOOS by Pearson correlation, changes in weight alone were not predictive of improvement in overall KOOS by multivariate linear mixed-effects regression. Instead, improvements in CAF and hsCRP were each independent predictors associated with significant improvements in overall KOOS at 2 years. Other studies have demonstrated a strong correlation between weight loss and improved patient reported knee pain and function scores in overweight and T2D patients [19]. The Look AHEAD trial demonstrated an intensive lifestyle intervention (ILI) involving diet/exercise education and supervision led to significant weight loss compared to usual care in a T2D patient population [43]. The study showed statistically significant improvement in Western Ontario and McMaster Universities Osteoarthritis Index (WOMAC) knee scores in participants as well as a decrease in overall inflammation as measured by hsCRP [21, 44]. The IDEA trial showed similar results in WOMAC scores and reported reduction in inflammation measured by IL-6 levels in an overweight and obese population without diabetes [16]. The current study confirms and builds upon these findings in that it demonstrates similar improvement in patient reported knee pain and function (KOOS) at 1 and 2 years as well as reaffirms a potential role of reduced inflammation, in this case measured by hsCRP. Our study also further demonstrates that CCI participants were not only able to achieve statistically significant improvement in knee pain and function but also approximately 46% of the CCI participants had a clinically meaningful improvement of over 10 points in total KOOS score [31]. Additionally, CCI participants were more likely to achieve this improvement than their UC counterparts. Achieving clinically meaningful improvements suggests that CCI participants may be more likely to increase physical activities [31], and hence achieve additional benefits from fitness associated with alleviating knee pain and improving knee function [19, 45].

While many studies have demonstrated improved knee pain with reduced body weight [15, 16, 19], this study is unique because, in addition to demonstrating a correlation with weight loss and improved overall KOOS score, it identifies central abdominal fat as the only anthropometric measure to predict improvement in total KOOS scores. This coincides with recent findings by Pan et al. noting a correlation between greater central obesity and increased knee pain among individuals with metabolic syndrome [46]. Furthermore, our results were in accordance with other studies that also assessed body composition indices [17, 47]. These studies revealed that changes in body fat or percentage body fat explained a greater proportion of variance in change in knee pain and function than weight loss alone [17, 47]. Given the fact that weight and adiposity are strongly correlated with each other, it is possible that studies reporting weight loss as a powerful predictor of knee improvement [15, 16, 19] may have also encountered reduction in body fat and central adiposity. Although a precise mechanistic link between central adipose tissue and knee pain has yet to be delineated, a number of mechanisms have been proposed for the relationship between obesity and pain [48]. Adipose tissue is also known to play an important role in producing proinflammatory cytokines. This presents a possible mechanism for increased knee pain and a potential explanation for the predictive nature of decreased abdominal fat and reduced serum hsCRP in improved knee pain and function [49, 50]. Additional studies are needed to further characterize the associations between adipose tissue, inflammatory markers, and knee pain.

The strengths of this study are prospective analysis, long term follow-up in a relatively large sample size, and extensive laboratory and anthropometric analysis at multiple time points. Among its potential weaknesses, this is a nonrandomized study performed in a single healthcare system which both represent potential areas for unintentional bias. Therefore, the interpretation of the primary findings was limited to the longitudinal change in total KOOS and its subscores in CCI and UC. The knee score assessment was also limited by the level of missingness in the data. There are potential unmeasured confounders that are not accounted for in the sensitivity analysis and the true missing data trajectory cannot be confirmed from the data. Additional exploratory aims were included to understand the impact of the intervention on knee pain and scores mainly for future study planning. Furthermore, there is an absence of information regarding radiographically confirmed diagnoses of OA in the study subjects. This challenges the direct applicability of our findings to patients with OA as we do not know which of the participants have radiographic OA and which do not. However, given that the causes of knee pain in this population with a high initial BMI are varied and often present in the absence of radiographically significant OA, this study does provide clinical application in that it demonstrates improved generic knee pain and function via a ketogenic diet without regard to an underlying diagnosis.

## Conclusions

A continuous care intervention including nutritional ketosis was associated with significant improvements in knee pain and function in patients with T2D. While these improvements were observed concurrently with weight loss, this study suggested that the change in knee function were possibly mediated by reduction in central adiposity and inflammation. The findings from this study prompt future research on the applicability of this intervention in radiographically confirmed OA patients.

## Supplementary Information

**Additional file 1: Supplementary Method Section. Supplementary Table 1.** Baseline characteristics of participants with knee pain in CCI and UC and comparison of completers and dropouts or missing data. **Supplementary Table 2.** Pattern-mixture model sensitivity analysis based on multiple imputation and different MNAR assumption for missing KOOS total and subscores data. **Supplementary Figure 1.** Patient flowchart from baseline to 2 years with knee score data.

## Data Availability

The datasets analyzed in the current study are available from the corresponding authors on reasonable request.

## Abbreviations

T2D: Type 2 diabetes
CCI: Continuous care intervention
UC: Usual care
OA: Osteoarthritis
KOOS: Knee Injury and Osteoarthritis Outcome Score
CAF: Central abdominal fat
hsCRP: High sensitivity C-reactive protein
IDEA: Intensive diet and exercise for arthritis
BMI: Body mass index
CLIA: Clinical laboratory improvement amendments
DXA: Dual X-ray absorptiometry
MIC: Minimal clinically important change
ILI: Intensive lifestyle intervention
WOMAC: Western Ontario and McMaster Universities Osteoarthritis Index
