# Supplementary material for: Continuous care intervention with carbohydrate restriction improves physical function of the knees among patients with type 2 diabetes: a non-randomized study
Source: BMC Musculoskelet Disord. 2022 Mar 29;23:297. doi: 10.1186/s12891-022-05258-0 (PMC8961996; doi:10.1186/s12891-022-05258-0)

**Supplementary Statistical Method**

**Sensitivity Analysis with Pattern Mixture Models**

- 1. Multiple imputation

The missing KOOS total and its’ individual subscale scores at each time points (baseline, 1 and 2 years) were imputed for both CCI and UC using a linear regression model under the missing at random (MAR) assumption. Baseline characteristics including age, gender, weight, and HbA1c were included as predictor in the model along with the available total KOOS and its’ individual subscale scores. A final 50 multiple imputed datasets were created from KOOS total and its’ individual subscale scores

- 1. Primary analysis

We then performed linear mixed effect models (LMMs) to estimate the within-group (CCI and UC) and between-group (CCI vs UC) differences in the KOOS total and its individual subscale scores on the 50 MI datasets. The LMMs included time effect, group (CCI vs UC) and time by group interactions. Several covariates were included in the analysis including age, sex, race, diabetes duration, baseline BMI and insulin use. A maximum likelihood approach was used to handle all available repeated measures in the analysis and an unstructured covariance was specified to account for correlations between the repeated measures. Fifty different LMM models were fitted, and the results were pooled using Rubin’s inference. The primary analysis covered the 1:1 scenario where both CCI and UC were imputed under MAR assumption.

- 1. Modification of MAR imputed values to create MNAR imputed values

To investigate different MNAR scenarios, we multiplied the original MAR imputed values from the primary analysis by a sensitivity parameter, δ to generate MNAR imputed values

(MNAR imputed values) = δ x (MAR imputed values)

For our KOOS assessment, we decided to use δ ranging from 5 to 10% difference between the observed values and missing values. The 50 MI KOOS total and its individual subscores for CCI and UC were modified using different MNAR situations, 0.9:1, 1:0.9, 0.9:0.95, 0.95:0.9, 0.9:0.9, 1:1.1 and 0.9:1.1. For example, scenario 0.9:1, the multiple imputed values in CCI were decreased 10% than the original imputed values while the original imputed values were maintained in UC. We also performed a conservative MNAR scenario 0.9:1.1, where the imputed values in CCI were assumed to be worse than the observed values (decreased 10%), while the imputed values in UC were assumed to have better outcome than the observed values (increased 10%). Each MNAR plausible scenarios includes 50 different datasets.

- 1. Final sensitivity analysis

For each MNAR scenario consisting of 50 datasets, LMMs were fitted in each dataset and the results were pooled using Rubin’s inference. As in primary analysis, we estimated within group (CCI and UC) and between-group (CCI vs UC) differences in the KOOS total and its’ individual subscores. For the final interpretation, we assessed the longitudinal change in the KOOS total and its individual subscores in CCI and UC for all the different MNAR scenarios.

**Supplementary Table 1**. Baseline characteristics of participants with knee pain in CCI and UC and comparison of completers and dropouts or missing data

|  | **All** | | **Completers with data** | | **Dropout or missing data** | | **Completers-Dropouts** |
| --- | --- | --- | --- | --- | --- | --- | --- |
|  | **N** | **Mean (SD)** | **N** | **Mean (SD)** | **N** | **Mean (SD)** | **Mean ± SE** |
| **Age (years)**   CCI T2D  UC T2D  CCI vs UC  Standardized difference | 173  69 | 54.27(7.81)  52.97(8.69)  1.30± 1.15  0.16 | 123  53 | 55.21(7.42)  52.09(8.86)  3.12±1.30* | 50  16 | 51.94(8.33)  55.88(7.62)  -3.93± 2.35 | 3.27±1.30*  -3.78±2.45 |
| **Diabetes duration (years)**    CCI T2D  UC T2D  CCI vs UC  Standardized difference | 173  69 | 8.77(7.59)  7.79(7.16)  0.99±1.15  0.13 | 123  53 | 8.15(7.24)  8.02(7.32)  0.13±1.22 | 50  16 | 10.32(8.27)  5.83(5.71)  4.49±3.48 | -2.17±1.27  2.19±3.11 |
| **Female (%)**   CCI T2D  UC T2D  CCI vs UC  Standardized difference | 173  69 | 70.0(46.0)  57.0(50.0)  13.4±6.7  0.27 | 123  53 | 67.0(47.0)  57.0(50.0)  10.9±8.1 | 50  16 | 76.0(43.1)  56.0(51.2)  19.7±14.2 | -8.5±7.4  0.4±14.4 |
| **Hemoglobin A1c (%)**   CCI T2D  UC T2D  CCI vs UC  Standardized difference | 173  69 | 7.59(1.45)  7.56(1.77)  -0.03±0.22  0.02 | 123  53 | 7.53(1.40)  7.70(1.93)  -0.17±0.29 | 50  16 | 7.73(1.54)  7.09(0.99)  0.65±0.41 | -0.20±0.24  0.61±0.36 |
| **Fasting glucose (mg/dL)**   CCI T2D  UC T2D  CCI vs UC  Standardized difference | 171  68 | 161.36(60.87)  154.01(74.12)  7.35±9.30  0.10 | 121  52 | 157.48(58.79)  161.25(81.41)  -3.77±12.49 | 50  16 | 170.76(65.30)  130.50(35.12)  40.26±17.12* | -13.28±10.21  30.75±14.30* |
| **Weight-clinic (kg)**  CCI T2D  UC T2D  CCI vs UC  Standardized difference | 169  67 | 120.31(27.6)  105.40(21.90)  14.92±3.77*  0.60 | 120  51 | 121.65(26.46)  106.81(22.83)  14.83±4.25* | 49  16 | 117.03(30.12)  100.88(18.60)  16.60±6.34* | 4.61±4.67  5.93±6.28 |
| **Central abdominal fat (kg)**   CCI T2D | 152 | 6.03(1.68) | 107 | 6.04(1.63) | 45 | 5.98(1.80) | -0.06±0.30 |
| **hs C-reactive protein (mg L^1^)**   CCI T2D  UC T2D  CCI vs UC  Standardized difference | 164  67 | 9.23(16.95)  8.93(8.72)  0.30±2.18  0.02 | 118  52 | 9.82(19.50)  9.00(8.92)  0.82±2.83 | 46  15 | 7.72(6.96)  8.69(8.28)  0.97±2.17 | 2.10±2.95  0.32±2.57 |
| **White blood cell (k/cumm)**   CCI T2D  UC T2D  CCI vs UC  Standardized difference | 172  68 | 7.28(1.89)  8.15(2.49)  -0.88±0.30*  -0.39 | 122  52 | 7.14(1.75)  8.20(2.38)  -1.06±0.37* | 50  16 | 7.60(2.19)  7.99(2.91)  -0.39±0.68 | -0.46±0.32  0.20±0.72 |
| **Total KOOS**  CCI T2D  UC T2D  CCI vs UC  Standardized difference | 141  50 | 68.36(22.27)  66.19(20.27)  2.16±3.58  0.10 | 103  37 | 67.25(23.10)  63.27(19.22)  3.98±4.25 | 39  13 | 71.26(19.93)  74.51(21.61)  -3.26±6.52 | -4.01±4.19  -11.25±6.40 |
| **KOOS Symptoms**  CCI T2D  UC T2D  CCI vs UC  Standardized difference | 152  51 | 71.47(21.01)  72.27(16.11)  -0.80±2.83  -0.04 | 107  38 | 70.27(21.05)  70.30(13.13)  -0.03±2.95 | 45  13 | 74.33(20.87)  78.02(22.39)  -3.70±6.68 | -4.05±3.73  -7.72±6.57 |
| **KOOS Pain**  CCI T2D  UC T2D  CCI vs UC  Standardized difference | 149  52 | 73.40(19.90)  69.81(21.93)  3.59±3.29  0.17 | 107  39 | 72.80(20.05)  66.72(20.85)  6.08±3.79 | 42  13 | 74.91(19.65)  79,06(23.31)  -4.15±6.52 | -2.11±3.63  -12.34±6.88 |
| **KOOS Activities of Daily Living**  CCI T2D  UC T2D  CCI vs UC  Standardized difference | 151  52 | 77.79(20.15)  73.43(21.95)  4.36±3.32  0.21 | 107  39 | 77.22(20.95)  70.53(22.62)  6.70±4.00 | 44  13 | 79.16(18.21)  82.13(17.80)  -2.96±5.72 | -1.94±3.62  -11.60±6.90 |
| **KOOS Sports and Recs**  CCI T2D  UC T2D  CCI vs UC  Standardized difference | 143  51 | 58.95(33.17)  58.92(28.62)  0.03±4.87  0.00 | 103  38 | 57.98(34.23)  57.63(29.12)  0.35±5.82 | 41  13 | 61.37(30.61)  62.69(27.81)  -1.32±9.54 | -3.39±6.15  -5.06±9.26 |
| **KOOS Quality of Life**  CCI T2D  UC T2D  CCI vs UC  Standardized difference | 150  52 | 59.68(28.23)  54.21(25.81)  5.47±4.45  0.20 | 106  39 | 58.43(29.10)  48.72(23.48)  9.71±5.19 | 44  13 | 62.69(26.09)  70.67(26.32)  -7.98±8.25 | -4.26±5.07  -21.96±7.75* |

Abbreviations: SD, standard deviation; SE, standard error; CCI, continuous care intervention; UC, usual care; T2D, type 2 diabetes; KOOS, knee osteoarthritis outcome scores. A significance level of P<0.008 ensures overall simultaneous significance of P < 0.05 over the 6 variables using Bonferroni correction.

Demographic and clinical characteristics’ assessment for total and its individual KOOS subscale scores were based on available data at baseline and 2 years. There were missingness in the knee data at baseline. Completers with data were those with available data at 2 years.

Standardized differences are differences in means or proportions adjusted by standard error. A standardized difference of greater than 0.10 is defined as imbalance.

**Supplementary Table 2**. Pattern-mixture model sensitivity analysis based on multiple imputation and different MNAR assumption for missing KOOS total and subscores data

|  |  | Baseline | One Year | Two Years |
| --- | --- | --- | --- | --- |
| Total KOOS |  |  |  |  |
| 1 CCI:1 UC MAR | CCI (1) | 69.4 (66.6, 72.2) | 79.3 (76.7,81.9) *** | 79.4 (76.7, 82.0) *** |
|  | UC (1) | 64.7 (60.2, 69.2) | 65.2 (60.9, 69.4) | 61.0 (56.7, 65.2) |
|  | CCI vs UC | 4.6 (-0.7, 10.0) | 14.1 (9.1, 19.1) *** | 18.4 (13.4, 23.5) *** |
| 0.9 CCI: 1 UC | CCI (0.9) | 68.0 (65.2, 70.8) | 69.6 (67.2, 72.0) | 76.1 (73.4, 78.7) *** |
|  | UC (1) | 64.7 (60.2, 69.2) | 65.2 (60.9, 69.4) | 61.0 (56.7, 65.2) |
|  | CCI vs UC | 3.3 (-2.0, 8,6) | 4.4 (-0.3, 9.1) | 15.1 (10.1, 20.1) *** |
| 1 CCI: 0.9 UC | CCI (1) | 69.4 (66.6, 72.2) | 79.3 (76.7,81.9) *** | 79.4 (76.7, 82.0) *** |
|  | UC (0.9) | 65.0 (60.5, 69.5) | 62.8 (58.7, 66.8) | 58.3 (54.1, 62.6) |
|  | CCI vs UC | 4.4 (-0.9, 9.7) | 16.5 (11.7, 21.3) *** | 21.1 (16.1, 26.1) *** |
| 0.9 CCI: 0.95 UC | CCI (0.9) | 68.0 (65.2, 70.8) | 69.6 (67.2, 72.0) | 76.1 (73.4, 78.7) *** |
|  | UC (0.95) | 64.2 (59.6, 68.7) | 63.4 (59.5, 67.3) | 59.9 (55.6, 64.2) |
|  | CCI vs UC | 3.8 (-1.5, 9.1) | 6.2 (1.7, 10.7) ** | 16.2 (11.2, 21.2) *** |
| 0.95 CCI: 0.9 UC | CCI (0.95) | 68.6 (65.8, 71.4) | 71.0 (68.6, 73.4) ** | 77.7 (75.0, 80.3) *** |
|  | UC (0.9) | 65.0 (60.5, 69.5) | 62.8 (58.7, 66.8) | 58.3 (54.1, 62.6) |
|  | CCI vs UC | 3.6 (-1.7, 8.9) | 8.2 (3.6, 12.8) *** | 19.4 (14.4, 24.4) *** |
| 0.9 CCI: 0.9 UC | CCI (0.9) | 68.0 (65.2, 70.8) | 69.6 (67.2, 72.0) | 76.1 (73.4, 78.7) *** |
|  | UC (0.9) | 65.0 (60.5, 69.5) | 62.8 (58.7, 66.8) | 58.3 (54.1, 62.6) |
|  | CCI vs UC | 3 (-2.3, 8.3) | 6.8 (2.2, 11.4) ** | 17.8 (12.8, 22.8) *** |
| 1 CCI: 1.1 UC | CCI (1) | 69.4 (66.6, 72.2) | 79.3 (76.7, 81.9) *** | 79.4 (76.7, 82.0) *** |
|  | UC (1.1) | 65.0 (60.5, 69.5) | 65.1 (61.1, 69.2) | 64.3 (60.0, 68.5) |
|  | CCI vs UC | 4.4 (-0.9, 9.7) | 14.2 (9.4, 19.0) *** | 15.1 (10.1, 20.1) *** |
| 0.9 CCI: 1.1 UC | CCI (0.9) | 68.0 (65.2, 70.8) | 69.6 (67.2, 72.0) | 76.1 (73.4, 78.7) *** |
|  | UC (1.1) | 65.0 (60.5, 69.5) | 65.1 (61.1, 69.2) | 64.3 (60.0, 68.5) |
|  | CCI vs UC | 3 (-2.3, 8.3) | 4.5 (-0.1, 9.1) | 11.8 (6.8, 16.8) *** |
| KOOS Symptoms | | | | |
| 1 CCI:1 UC MAR | CCI (1) | 72.8 (69.9, 75.6) | 79.8 (77.1, 82.6) *** | 80.3 (77.2, 83.5) *** |
|  | UC (1) | 71.2 (66.2, 76.3) | 70.2 (65.0, 75.4) | 65.8 (59.0, 72.7) |
|  | CCI vs UC | 1.6 (-3.5, 7.0) | 9.6 (3.3, 13.1) *** | 14.5 (10.0, 21.6) *** |
| 0.9 CCI: 1 UC | CCI (0.9) | 71.9 (69.1, 74.7) | 78.8 (76.0, 81.5) *** | 77.2 (74.1, 80.3) ** |
|  | UC (1) | 71.2 (66.2, 76.3) | 70.2 (65.0, 75.4) | 65.8 (59.0, 72.7) |
|  | CCI vs UC | 0.7 (-4.7, 6.2) | 8.6 (3.1, 14.1) *** | 11.4 (4.9, 17.9) *** |
| 1 CCI: 0.9 UC | CCI (1) | 72.8 (69.9, 75.6) | 79.8 (77.1, 82.6) | 80.3 (77.2, 83.5) |
|  | UC (0.9) | 69.2 (64.3, 74.2) | 68.1 (63.0, 73.2) | 62.7 (56.3, 69.1) |
|  | CCI vs UC | 3.6 (-1.9, 9.1) | 11.7 (6.3, 17.1) *** | 17.6 (11.2, 24.1) *** |
| 0.9 CCI: 0.95 UC | CCI (0.9) | 71.9 (69.1, 74.7) | 78.8 (76.0, 81.5) *** | 77.2 (74.1, 80.3) ** |
|  | UC (0.95) | 70.4 (65.3, 75.4) | 69.3 (64.1, 74.4) | 64.4 (57.8, 71.0) |
|  | CCI vs UC | 1.5 (-4.0, 14.9) | 9.5 (4.1, 14.9) *** | 12.8 (6.4, 19.2) *** |
| 0.95 CCI: 0.9 UC | CCI (0.95) | 72.3 (69.5, 75.2) | 79.3 (76.6, 82.0) *** | 78.7 (75.7, 81.8) *** |
|  | UC (0.9) | 69.2 (64.3, 74.2) | 68.1 (63.0, 73.2) | 62.7 (56.3, 69.1) |
|  | CCI vs UC | 3.1 (-2.4, 8.6) | 11.2 (5.8, 16.6) *** | 16.0 (9.7, 22.3) *** |
| 0.9 CCI: 0.9 UC | CCI (0.9) | 71.9 (69.1, 74.7) | 78.8 (76.0, 81.5) *** | 77.2 (74.1, 80.3) ** |
|  | UC (0.9) | 69.2 (64.3, 74.2) | 68.1 (63.0, 73.2) | 62.7 (56.3, 69.1) |
|  | CCI vs UC | 2.7 (-2.7, 8.1) | 10.7 (5.3, 16.1) *** | 14.5 (8.1, 20.9) *** |
| 1 CCI: 1.1 UC | CCI (1) | 72.8 (69.9, 75.6) | 79.8 (77.1, 82.6) | 80.3 (77.2, 83.5) |
|  | UC (1.1) | 73.3 (68.0, 78.5) | 72.2 (66.9, 77.5) | 68.9 (61.6, 76.2) |
|  | CCI vs UC | -0.5 (-6.1, 5.1) | 7.6 (2.1, 13.1) ** | 11.4 (4.6, 18.2) *** |
| 0.9 CCI: 1.1 UC | CCI (0.9) | 71.9 (69.1, 74.7) | 78.8 (76.0, 81.5) *** | 77.2 (74.1, 80.3) ** |
|  | UC (1.1) | 73.3 (68.0, 78.5) | 72.2 (66.9, 77.5) | 68.9 (61.6, 76.2) |
|  | CCI vs UC | -1.4 (-6.9, 4.1) | 6.6 (1.1, 12.1) | 8.3 (1.6, 15.0) ** |
| KOOS Pain | | | | |
| 1 CCI:1 UC MAR | CCI (1) | 74.7 (71.9, 77.5) | 83.5 (80.6, 86.3) *** | 84.0 (80.7, 87.2) *** |
|  | UC (1) | 68.6 (63.9, 73.4) | 67.4 (63.0, 71.7) | 67.5 (60.9, 74.0) |
|  | CCI vs UC | 6.1 (0.6, 11.3) | 16.1 (11.2, 21.4) *** | 165 (13.1, 23.5) *** |
| 0.9 CCI: 1 UC | CCI (0.9) | 73.6 (70.8, 76.5) | 80.5 (77.6, 83.3) *** | 80.7 (77.5, 83.9) *** |
|  | UC (1) | 68.6 (63.9, 73.4) | 67.4 (63.0, 71.7) | 67.5 (60.9, 74.0) |
|  | CCI vs UC | 5.0 (-0.4, 10.4) | 13.1 (7.8, 18.4) *** | 13.2 (6.7, 19.7) *** |
| 1 CCI: 0.9 UC | CCI (1) | 74.7 (71.9, 77.5) | 83.5 (80.6, 86.3) *** | 84.0 (80.7, 87.2) *** |
|  | UC (0.9) | 66.9 (62.2, 71.6) | 66.2 (61.8, 70.6) | 64.3 (58.1, 70.5) |
|  | CCI vs UC | 7.8 (2.5, 13.1) ** | 17.3 (12.0, 22.6) *** | 19.7 (13.2, 26.2) *** |
| 0.9 CCI: 0.95 UC | CCI (0.9) | 73.6 (70.8, 76.5) | 80.5 (77.6, 83.3) *** | 80.7 (77.5, 83.9) *** |
|  | UC (0.95) | 67.9 (63.1, 72.6) | 66.9 (62.5, 71.3) | 66.0 (59.6, 72.4) |
|  | CCI vs UC | 5.7 (0.3, 11.1) | 13.6 (8.3, 18.9) *** | 14.7 (8.2, 21.2) *** |
| 0.95 CCI: 0.9 UC | CCI (0.95) | 74.2 (71.3, 77.0) | 82.0 (79.1, 84.8) *** | 82.3 (79.1, 85.4) *** |
|  | UC (0.9) | 66.9 (62.2, 71.6) | 66.2 (61.8, 70.6) | 64.3 (58.1, 70.5) |
|  | CCI vs UC | 7.3 (1.9, 12.7) | 15.8 (10.5, 21.1) *** | 18.0 (11.7, 24.3) *** |
| 0.9 CCI: 0.9 UC | CCI (0.9) | 73.6 (70.8, 76.5) | 80.5 (77.6, 83.3) *** | 80.7 (77.5, 83.9) *** |
|  | UC (0.9) | 66.9 (62.2, 71.6) | 66.2 (61.8, 70.6) | 64.3 (58.1, 70.5) |
|  | CCI vs UC | 6.7 (1.3, 12.1) | 14.3 (9.0, 19.6) *** | 16.4 (10.0, 22.8) *** |
| 1 CCI: 1.1 UC | CCI (1) | 74.7 (71.9, 77.5) | 83.5 (80.6, 86.3) *** | 84.0 (80.7, 87.2) *** |
|  | UC (1.1) | 70.4 (65.5, 75.2) | 68.6 (64.2, 73.0) | 70.7 (63.7, 77.7) |
|  | CCI vs UC | 4.3 (-1.1, 9.7) | 14.9 (9.6, 20.2) *** | 13.3 (6.5, 20.1) *** |
| 0.9 CCI: 1.1 UC | CCI (0.9) | 73.6 (70.8, 76.5) | 80.5 (77.6, 83.3) *** | 80.7 (77.5, 83.9) *** |
|  | UC (1.1) | 70.4 (65.5, 75.2) | 68.6 (64.2, 73.0) | 70.7 (63.7, 77.7) |
|  | CCI vs UC | 3.2 (-2.3, 8.7) | 11.9 (6.6, 17.2) *** | 10.0 (3.3, 16.7) *** |
| KOOS ADL | | | | |
| 1 CCI:1 UC MAR | CCI (1) | 78.9 (76.1, 81.7) | 87.1 (84.6, 89.5) *** | 87.4 (84.5, 90.2) *** |
|  | UC (1) | 71.9 (67.2, 76.5) | 70.9 (67.1, 74.7) | 64.5 (59.8, 69.3) |
|  | CCI vs UC | 7.0 (1.8, 12.3) | 16.2 (12.0, 21.0) *** | 22.9 (17.9, 28.7) *** |
| 0.9 CCI: 1 UC | CCI (0.9) | 77.9 (75.1, 80.6) | 84.1 (81.6, 86.6) *** | 83.9 (81.1, 86.8) *** |
|  | UC (1) | 71.9 (67.2, 76.5) | 70.9 (67.1, 74.7) | 64.5 (59.8, 69.3) |
|  | CCI vs UC | 6.0 (0.7, 11.3) | 13.2 (8.6, 17.8) *** | 19.4 (14.0, 24.8) *** |
| 1 CCI: 0.9 UC | CCI (1) | 78.9 (76.1, 81.7) | 87.1 (84.6, 89.5) *** | 87.4 (84.5, 90.2) *** |
|  | UC (0.9) | 70.0 (65.4, 74.7) | 69.6 (65.8, 73.5) | 61.5 (56.8, 66.2) |
|  | CCI vs UC | 8.9 (3.6, 14.2) *** | 17.5 (13.0, 22.1) *** | 25.9 (20.4, 31.3) *** |
| 0.9 CCI: 0.95 UC | CCI (0.9) | 77.9 (75.1, 80.6) | 84.1 (81.6, 86.6) *** | 83.9 (81.1, 86.8) *** |
|  | UC (0.95) | 71.1 (66.4, 75.8) | 70.4 (66.5, 74.3) | 63.2 (58.4, 67.9) |
|  | CCI vs UC | 6.8 (1.5, 12.1) | 13.7 (9.0, 18.3) *** | 20.7 (15.3, 26.1) *** |
| 0.95 CCI: 0.9 UC | CCI (0.95) | 78.4 (75.6, 81.1) | 85.6 (83.1, 88.1) *** | 85.6 (82.8, 88.5) *** |
|  | UC (0.9) | 70.0 (65.4, 74.7) | 69.6 (65.8, 73.5) | 61.5 (56.8, 66.2) |
|  | CCI vs UC | 8.4 (3.1, 13.7) ** | 16.0 (11.4, 20.6) *** | 24.1 (18.7, 29.5) *** |
| 0.9 CCI: 0.9 UC | CCI (0.9) | 77.9 (75.1, 80.6) | 84.1 (81.6, 86.6) *** | 83.9 (81.1, 86.8) *** |
|  | UC (0.9) | 70.0 (65.4, 74.7) | 69.6 (65.8, 73.5) | 61.5 (56.8, 66.2) |
|  | CCI vs UC | 7.9 (2.6, 13.2) ** | 14.5 (9.9, 19.1) *** | 22.4 (17.0, 27.8) *** |
| 1 CCI: 1.1 UC | CCI (1) | 78.9 (76.1, 81.7) | 87.1 (84.6, 89.5) *** | 87.4 (84.5, 90.2) *** |
|  | UC (1.1) | 73.7 (68.9, 78.4) | 72.1 62.7, 72.5) | 67.6 (62.7, 72.5) |
|  | CCI vs UC | 5.2 (-0.2, 10.6) | 15.0 (10.0, 20.0) *** | 19.8 (14.3, 25.3) *** |
| 0.9 CCI: 1.1 UC | CCI (0.9) | 77.9 (75.1, 80.6) | 84.1 (81.6, 86.6) *** | 83.9 (81.1, 86.8) *** |
|  | UC (1.1) | 73.7 (68.9, 78.4) | 72.1 62.7, 72.5) | 67.6 (62.7, 72.5) |
|  | CCI vs UC | 4.2 (-1.1, 9.5) | 12.0 (7.0, 17.0) *** | 16.3 (10.8, 21.8) *** |
| KOOS Sport.Rec | | | | |
| 1 CCI:1 UC MAR | CCI (1) | 58.9 (54.2, 63.7) | 73.1 (68.7, 77.5) *** | 71.7 (66.4, 77.1) *** |
|  | UC (1) | 60.8 (53.2, 68.4) | 61.4 (54.6, 68.1) | 54.9 (46.3, 63.5) |
|  | CCI vs UC | -1.9 (-10.3, 6.6) | 11.7 (3.2, 19.6) ** | 16.8 (7.7, 25.9) *** |
| 0.9 CCI: 1 UC | CCI (0.9) | 58.1 (53.4, 62.7) | 70.6 (66.3, 74.9) *** | 68.9 (63.7, 74.0) *** |
|  | UC (1) | 60.8 (53.2, 68.4) | 61.4 (54.6, 68.1) | 54.9 (46.3, 63.5) |
|  | CCI vs UC | -2.7 (-11.4, 6.1) | 9.2 (1.2, 17.2) | 14.0 (4.2, 23.8) *** |
| 1 CCI: 0.9 UC | CCI (1) | 58.9 (54.2, 63.7) | 73.1 (68.7, 77.5) *** | 71.7 (66.4, 77.1) *** |
|  | UC (0.9) | 59.1 (51.5, 66.6) | 60.3 (53.5, 67.0) | 52.4 (44.0, 60.7) |
|  | CCI vs UC | -0.2 (-9.1, 8.7) | 12.8 (4.6, 21.0) *** | 19.3 (9.3, 29.3) *** |
| 0.9 CCI: 0.95 UC | CCI (0.9) | 58.1 (53.4, 62.7) | 70.6 (66.3, 74.9) *** | 68.9 (63.7, 74.0) *** |
|  | UC (0.95) | 59.9 (52.4, 67.4) | 60.8 (54.2, 67.5) | 53.6 (45.3, 61.9) |
|  | CCI vs UC | -1.8 (-10.6, 7.0) | 9.8 (1.8, 17.8) | 15.3 (5.6, 25.0) *** |
| 0.95 CCI: 0.9 UC | CCI (0.95) | 58.5 (53.8, 63.2) | 71.8 (67.5, 76.2) *** | 70.3 (65.1, 75.5) *** |
|  | UC (0.9) | 59.1 (51.5, 66.6) | 60.3 (53.5, 67.0) | 52.4 (44.0, 60.7) |
|  | CCI vs UC | -0.6 (-9.4, 8.2) | 11.5 (3.4, 19.6) ** | 17.9 (8.1, 27.7) *** |
| 0.9 CCI: 0.9 UC | CCI (0.9) | 58.1 (53.4, 62.7) | 70.6 (66.3, 74.9) *** | 68.9 (63.7, 74.0) *** |
|  | UC (0.9) | 59.1 (51.5, 66.6) | 60.3 (53.5, 67.0) | 52.4 (44.0, 60.7) |
|  | CCI vs UC | -1.0 (-9.8, 7.8) | 10.3 (2.3, 18.3) | 16.5 (6.8, 26.2) *** |
| 1 CCI: 1.1 UC | CCI (1) | 58.9 (54.2, 63.7) | 73.1 (68.7, 77.5) *** | 71.7 (66.4, 77.1) *** |
|  | UC (1.1) | 62.6 (54.9, 70.3) | 62.4 (55.7, 69.2) | 57.4 (48.6, 66.2) |
|  | CCI vs UC | -3.7 (-12.7, 5.3) | 10.7 (2.5, 18.9) | 14.3 (4.2, 24.4) ** |
| 0.9 CCI: 1.1 UC | CCI (0.9) | 58.1 (53.4, 62.7) | 70.6 (66.3, 74.9) *** | 68.9 (63.7, 74.0) *** |
|  | UC (1.1) | 62.6 (54.9, 70.3) | 62.4 (55.7, 69.2) | 57.4 (48.6, 66.2) |
|  | CCI vs UC | -4.5 (-13.3, 4.3) | 8.2 (0.2, 16.2) | 11.5 (1.6, 21.4) ** |
| KOOS QoL | | | | |
| 1 CCI:1 UC MAR | CCI (1) | 61.7 (57.9, 65.5) | 73.0 (69.1, 76.9) *** | 73.6 (69.6, 77.6) *** |
|  | UC (1) | 50.9 (44.3, 57.4) | 55.9 (49.9, 61.8) | 52.1 (44.7, 59.5) |
|  | CCI vs UC | 10.8 (4.1, 18.6) ** | 17.1 (10.6, 24.7) *** | 21.5 (14.9, 27.9) *** |
| 0.9 CCI: 1 UC | CCI (0.9) | 60.8 (57.0, 64.6) | 70.5 (66.7, 74.4) *** | 70.7 (66.8, 74.6) *** |
|  | UC (1) | 50.9 (44.3, 57.4) | 55.9 (49.9, 61.8) | 52.1 (44.7, 59.5) |
|  | CCI vs UC | 9.9 (2.6, 17.2) ** | 14.6 (7.4, 21.8) *** | 18.6 (10.9, 26.3) *** |
| 1 CCI: 0.9 UC | CCI (1) | 61.7 (57.9, 65.5) | 73.0 (69.1, 76.9) *** | 73.6 (69.6, 77.6) *** |
|  | UC (0.9) | 49.7 (43.3, 56.1) | 54.9 (48.9, 60.9) | 49.6 (42.5, 56.8) |
|  | CCI vs UC | 12.0 (4.8, 26.0) *** | 18.1 (10.9, 25.3) *** | 24.0 (16.2, 31.8) *** |
| 0.9 CCI: 0.95 UC | CCI (0.9) | 60.8 (57.0, 64.6) | 70.5 (66.7, 74.4) *** | 70.7 (66.8, 74.6) *** |
|  | UC (0.95) | 50.4 (43.9, 56.9) | 55.5 (49.6, 61.5) | 51.0 (43.7, 58.3) |
|  | CCI vs UC | 10.4 (3.1, 17.7) ** | 15.0 (7.8, 22.2) *** | 19.7 (12.0, 27.4) *** |
| 0.95 CCI: 0.9 UC | CCI (0.95) | 61.2 (57.4, 65.0) | 71.8 (67.9, 75.6) *** | 72.1 (68.2, 76.0) *** |
|  | UC (0.9) | 49.7 (43.3, 56.1) | 54.9 (48.9, 60.9) | 49.6 (42.5, 56.8) |
|  | CCI vs UC | 11.5 (4.3, 18.7) ** | 16.9 (9.7, 24.1) *** | 22.5 (14.9, 30.1) *** |
| 0.9 CCI: 0.9 UC | CCI (0.9) | 60.8 (57.0, 64.6) | 70.5 (66.7, 74.4) *** | 70.7 (66.8, 74.6) *** |
|  | UC (0.9) | 49.7 (43.3, 56.1) | 54.9 (48.9, 60.9) | 49.6 (42.5, 56.8) |
|  | CCI vs UC | 11.1 (3.9, 18.3) ** | 15.6 (8.4, 22.8) *** | 21.1 (13.5, 28.7) *** |
| 1 CCI: 1.1 UC | CCI (1) | 61.7 (57.9, 65.5) | 73.0 (69.1, 76.9) *** | 73.6 (69.6, 77.6) *** |
|  | UC (1.1) | 52.0 (45.4, 58.6) | 56.9 (50.9, 62.8) | 54.6 (46.8, 62.3) |
|  | CCI vs UC | 9.7 (2.4, 17.0) | 16.1 (8.9, 23.3) *** | 19.0 (11.0, 27.0) *** |
| 0.9 CCI: 1.1 UC | CCI (0.9) | 60.8 (57.0, 64.6) | 70.5 (66.7, 74.4) *** | 70.7 (66.8, 74.6) *** |
|  | UC (1.1) | 52.0 (45.4, 58.6) | 56.9 (50.9, 62.8) | 54.6 (46.8, 62.3) |
|  | CCI vs UC | 8.8 (1.5, 16.1) | 13.6 (6.4, 20.8) *** | 16.1 (8.2, 24.0) *** |

***Note*.** Ns for continuous care intervention =173 and Ns for usual care=69 for each MAR and MNAR scenarios. Any missing data at each assessment timepoints were multiple imputed 50 times and modified using the delta parameter. If listed as 1: primary imputed values under missing at random assumption; if listed 0.95: primary imputed values estimated 5% lower; 0.90: primary imputed values estimated 10% lower; 1.10: primary imputed values estimated 10% higher either in CCI or UC.

Unless otherwise noted, estimates reported were obtained from linear mixed-effects models which provide adjusted means and mean changes, controlling for baseline age, sex, race, body mass index, and insulin use for each dataset. The final estimates were pooled using Rubin’s rules resulting in an intent-to-treat analysis. A significance level of P<0.008 ensures overall simultaneous significance of P < 0.05 over the 6 variables using Bonferroni correction. *** p<0.001, **p<0.01

**Abbreviations:** T2D, type 2 diabetes; CI, confidence interval; CCI, continuous care intervention; UC, usual care; KOOS, knee osteoarthritis outcome scores; MAR, missing at random; MNAR, missing not at random

**Supplementary Figure 1.** Patient flowchart from baseline to 2 years with knee score data


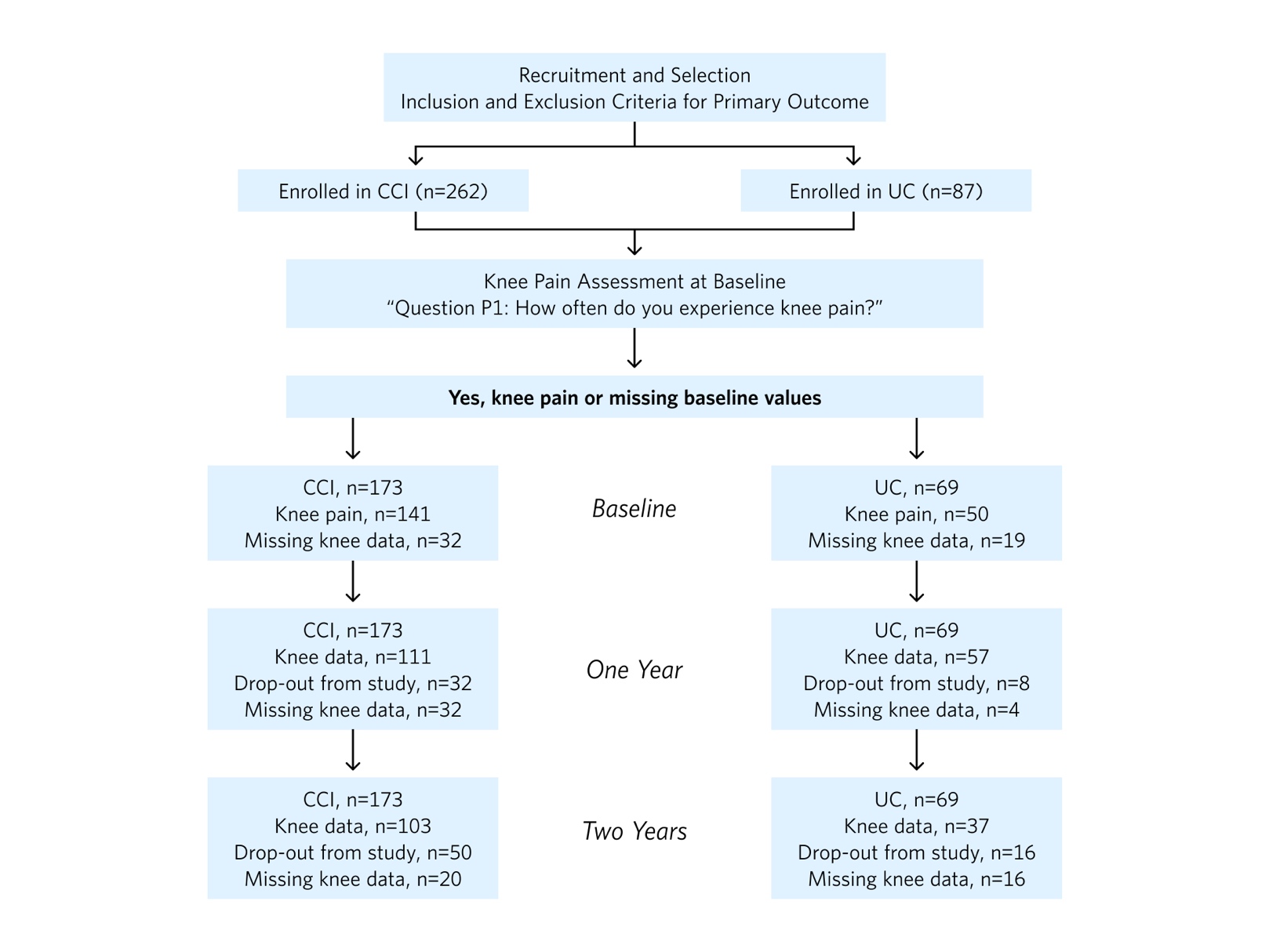

Supplement: Supplementary file 1 — Additional file 1: Supplementary Method Section. Supplementary Table 1. Baseline characteristics of participants with knee pain in CCI and UC and comparison of completers and dropouts or missing data. Supplementary Table 2. Pattern-mixture model sensitivity analysis based on multiple imputation and different MNAR assumption for missing KOOS total and subscores data. Supplementary Figure 1. Patient flowchart from baseline to 2 years with knee score data. [file 12891_2022_5258_MOESM1_ESM.docx]
